# ReNaApp: increasing the long-term effects of oncological rehabilitation through an application after medical rehabilitation (ReNaApp): a quasi-randomized longitudinal study of prospective design

**DOI:** 10.1186/s12913-020-05248-9

**Published:** 2020-05-06

**Authors:** Mercedes Rutsch, Nicole Jochems, Andreas Schrader, Iris Brandes, Lisa Weier, Ruth Deck

**Affiliations:** 1 Institute for Social Medicine and Epidemiology, Department Rehabilitation Sciences, University of Lübeck, Ratzeburger Allee 160, 23562 Lübeck, Germany; 2 Institute for Multimedia and Interactive Systems, University of Lübeck, Lübeck, Germany; 3 Institute of Telematics, University of Lübeck, Lübeck, Germany; 4 Institute for Epidemiology, Department Rehabilitation Sciences, Social Medicine and Health System Research, Hannover Medical School, Hannover, Germany; 5 Institute for Social Medicine and Epidemiology, University of Lübeck, Lübeck, Germany

**Keywords:** Breast cancer, Rehabilitation, Oncological rehab, Quality of life, Participation, Physical activity, Mobile application

## Abstract

**Background:**

Breast cancer is the most common malignant disease in women. Compared with other cancer types, breast cancer has a higher survival rate. The majority of breast cancer patients are overstrained to implement cancer-specific recommendations relating to changes in health behaviour. Numerous epidemiological studies have shown a positive correlation between physical activity and quality of life as well as the course of disease during and after breast cancer treatment. However, many patients have difficulties integrating physical activity into their everyday lives due to cancer symptoms. To develop physical activity into a daily routine, an aftercare programme for breast cancer patients will be developed. In particular, the programme is structured in terms of the validated concept “Neues Credo”. The basic concept is converted into a mobile application.

**Methods:**

The study sample includes *n* = 740 rehabilitants (370 for the intervention group and for the control group) from five different rehab clinics in Northern Germany. The evaluation is as follows: a) Quasi-randomized, prospective longitudinal study (sequential study design). The intervention group receives a mobile application after rehabilitation, and the control group receives treatment as usual. The study evaluation is carried out through a questionnaire at three stages (at the beginning of the rehabilitation, at the end of the rehabilitation, and after 12 months). b) Qualitative analysis of interviews and focus groups in terms of feasibility and acceptance. c) Formative evaluation of the app.

**Discussion:**

Above all, the aftercare programme ReNaApp increases the long-term effects of oncological rehabilitation. By documenting physical activity in ReNaApp, rehabilitants become more motivated to engage in physical activity in their everyday lives. Currently, there is no scientifically evaluated app for breast cancer patients in the German language. Thus, ReNaApp ensures an aftercare treatment for breast cancer patients with high-quality performance regardless of their place of residence. By adopting a participatory approach and a user-centred design, ReNaApp corresponds to the demands of the rehabilitants.

**Trial registration:**

German Register of Clinical Trials, www.drks.de.

Identifier: DRKS00019017; Registered: November 7th, 2019.

Date and version identifier: April 17th 2020; vesion 2.

## Background

With an incidence of 69,000 per year, breast cancer is one of the most common cancers that affect women in Germany [1]. In particular, breast cancer affects one in eight women, and three out of 10 are younger than 55 years old at the time of diagnosis [2]. During recovery, women are faced with symptoms and side effects, including fatigue, weight increase, decrease in muscle strength and bone density, pain, fear and depression, reduction of social and work-related participation and reduction of quality of life [3].

A number of authors have shown that physical activity is one of the most successful strategies in the course of an oncological disease. Physical activities increase the sense of well-being, quality of life and survival rate after cancer [3–6]. Moreover, the ability to work develops with physical activity [7]. Above all, the ability to work plays a crucial role for patients because it is connected with the return to normality. As a result, self-confidence, mental health and quality of life increase steadily [8, 9]. Nevertheless, given the symptoms, e.g., fatigue, weight gain or depression, it is difficult to implement physical activity in everyday life [10].

Medical rehabilitation is one opportunity to initiate an active lifestyle after breast cancer [11, 12]. To transfer the already-gained physical activity from rehab into the domestic environment, rehabilitants need an adequate aftercare programme [11, 12]. Regarding the current legal situation, an aftercare programme for breast cancer patients cannot be offered in Germany. There is, indeed, the chance of rehabilitation sports or functional training, but these often fail due to structural and local conditions.

Although there is no aftercare programme for breast cancer patients, an effective approach for other diseases has been developed. Further studies validated the aftercare concept called “Neues Credo”, which introduces and maintains physical activity. The aim of “Neues Credo” is to increase the physical activity of rehabilitants and help them maintain this increased engagement for long periods [13]. For instance, the concept has been evaluated in clinical trials with orthopaedic and psychosomatic patients and demonstrated long-term effects for rehabilitants [14, 15]. An analysis of a current study with cardiology rehabilitants supposes further success.

The aftercare programme “Neues Credo” contains a complex intervention. First, the rehabilitants obtain coping skills together with other patients during rehabilitation. Simultaneously, the rehab clinics provide an educated aftercare agent whose task is to motivate the patients during and after rehab. In this context, the agents support rehabilitants in achieving their individual goals and maintaining physical activity in everyday life.

While the concept “Neues Credo” only consists of a paper version, mobile applications for healthcare have been increasingly established in recent years. A reason for that is the rising importance of smartphones in the current society. For example, in January 2017, more than 54 million people over the age of 14 owned a smartphone in Germany. In other words, 62% of Germans use a smartphone [16]. In addition to the increase in smartphone users, the use of mobile applications increased up to 52% among individuals in the age range of 40–49 years and 34% among individuals in the age range of 50–59 years, tending upwards [17]. In the present study, “Neues Credo” can for the first time be implemented as a smartphone app, called ReNaApp. This app considers the needs of women with breast cancer.

Recently, there has been a growing interest in mobile health (mhealth) applications that support healthcare services through smartphone/mobile terminals [18]. Actually, the most common play stores have a selection of over 100,000 health apps [19, 20]. Approximately 29% of Germans have installed healthcare apps on their smartphone [19, 20]. However, most freely available apps have a lower quality than apps requiring payment [18, 21]. Many applications are developed outside of the primary health care sector [19, 20] and, unfortunately, lack a theoretical basis. Moreover, there are usually no proof-of-concepts on the efficiency of those tools [18–21].

Due to the great potential of mobile applications in healthcare, a need for evidence-based and high-quality applications arises [18, 21]. Previous reviews emphasized the efficiency of such applications [22–25]. Additionally, randomized clinical trials (RCTs) detected positive effects for patients with different medical indications [26]. Finally, one study observed successful results of a mobile application for breast cancer patients [27]. In conclusion, there is a lack of evidence of high-quality RCTs in this field of research [22–25].

The aftercare programme “Neues Credo” complies with the needed theoretical foundation [28] and has shown significant efficiency in different indications [14, 15]. The present study will modify the concept from paper-based materials into a mobile application and adapt it to the needs of women with breast cancer.

### Aims

This study aimed to integrate more physical activity into the life of breast cancer patients after rehab. (1) During rehabilitation, breast cancer patients are introduced to physical activities, define goals for the time after rehab and finally develop a plan for their implementation. (2) After rehabilitation, the patients install the ReNaApp, which includes an exercise diary, to reach their individual goals. In addition to the diary, the app encompasses tools for individual feedback, motivating functions and a comprehensive information area. (3) Consequently, the aftercare programme ReNaApp increases the physical activity of rehabilitants because of their greater motivation to permanently maintain the achievements of rehabilitation. More specifically, expectations in terms of improved social and professional participation, quality of life, return to work and general health are forecast over a period of 12 months.

### Intervention

Initially, each of the participating clinics chooses one aftercare agent for the whole duration of the project. The agents will be trained concerning the recruiting strategy, the philosophy of the aftercare programme, and the handling of the ReNaApp. During rehabilitation, the intervention group (IG) will be supported by them.

During rehabilitation, the participants of the study will receive a paper-based observation book. The observation book targets good preparation for the time after rehabilitation by the following three tasks: 1) self-monitoring: Which types of physical activity do I enjoy, and which physical activities make me feel good? 2) Which suitable physical activities exist, and which would I like to try? 3) What kind of physical activities are near my home?

At the end of rehab, the patients will be invited to individually plan their physical activity according to their observation books: Which type of physical activities will I integrate and when? Therefore, the rehabilitants join a group for an hour to discuss the observation book with other rehabilitants and therapists. The therapists especially advise the preparation of personal plans for implementation and compliance. Within the group hour, all patients write a letter to themselves, which is sent by the aftercare agents to every patient 3 months post-rehab.

As already mentioned, the aftercare programme encompasses a mobile application. ReNaApp is available on the web page of the involved clinics. During the class hour, the aftercare agents introduce the intervention group to downloading and using the mobile application. After logging into the app, the patients can use the application during the following 12 months. Related conversations with rehabilitants show that the use of a mobile application can expect high acceptance.

Furthermore, ReNaApp contains additional features that are not included in “Neues Credo”. First, the app automatically generates feedback in relation to the executed physical activities. Replacement of the classic paper-based activity diary with ReNaApp, involves changes in both the medium and the time interval of the feedback. Concerning the participants’ activities, the type and frequency of notifications are adapted by the app. In the aftercare period, the rehabilitants receive motivational letters from their aftercare agents at regular intervals (after 3, 6 and 9 months). In particular, the letters refer to maintaining physical activity or encouraging the rehabilitants to be more active.

In addition, the control group obtains the standardized therapy during rehabilitation as well as in aftercare. During rehab, the patients supply care from their clinic regarding the existing standards. The Sixth Social Code implies that only functional training or rehabilitation sports are offered to oncological rehabilitants after rehab.

### Mobile application

All rehabilitants of the intervention group document their physical activity in the exercise diary in ReNaApp. This flexible procedure ensures that the quality and functionality of the mobile application works permanently.

The currently available smartphones have a high degree of heterogeneity in brand, performance, display size, and software, with diverse operating systems. To include the largest possible number of smartphone users, ReNaApp uses frameworks and APIs that ensure compatibility with both iOS and Android operating systems, which include the vast majority of potential users’ devices. Because of impressive graphical frameworks and innovation in web technology, ReNaApp will be designed as a progressive web application (PWA) with a responsive design for automatic screen adaption [29, 30] and complete offline availability. This also ensures a convenient way for installing the app while allowing the project owners to keep complete control of the distribution of the software.

The user-centred design approach DIN EN ISSO 9241–210 [31] ensures high usability and acceptance for mobile applications. In particular, breast cancer patients are involved throughout the whole process. First, the user-centred design approach includes the planning of the design process as well as the empirical specification of usage requirements. The main stage searches for layout solutions for fulfilment of the usage requirements. In particular, the second phase encompasses the development and design of the human-technology interface of ReNaApp. These solutions will be tested and revised in terms of an iterative formative evaluation. Following a subsequent evaluation in the laboratory, the system will be optimized if necessary.

Like in the exercise diaries on paper, the patients will document their intentions regarding physical activity in ReNaApp. The documentation includes a selection of different types of physical activities, as well as custom activities. Furthermore, ReNaApp reminds the patients of their defined intentions regarding physical activity. Following a planned physical activity, the rehabilitants are requested to document whether they stuck to their plan and practiced physical activity for the full amount of time projected. Moreover, the patients record barriers, promoting factors and alternatives in terms of implementation problems.

An alternative option for tracking physical activity is to connect health tracker devices and integrate their data. Previous research shows that approximately 74% of all these devices belong to either Fitbit or Xiaomi [32]. Fitbit enables the access of collected data using interfaces [33–35] only by connecting to the Fitbit cloud service but not locally via Bluetooth [34]. In fact, most manufacturers give no guarantee for the support of collecting private data directly from end user devices without a cloud connection. Except for some reverse engineering approaches, most of the fitness devices cannot be connected with arbitrary apps via Bluetooth.

The second market leader, Xiaomi, only enables unofficial opportunities to transfer data via Bluetooth [36, 37]. Two recent tests by the paper c’t [33, 38] examined alternative fitness trackers without cloud connections, but these are not represented in the mentioned distribution statistics. Consequently, to integrate fitness trackers, it is necessary to obtain the approval of the users for the initial connection between the devices. This option exists in almost every common fitness tracker. Considering privacy concerns, this study does not pursue the mentioned approach.

To avoid this problem, health-middleware services from both operation systems (Android, iOS), i.e., Google Fit [39] and Apple Health Kit [36], can be used. With these, it is possible to integrate any device by the detour of a middleware approach. Because these are cloud services, privacy concerns with respect to data transfer, processing and storing emerge. Because of these concerns, middleware solutions will not be used.

In addition to the data protection and technical difficulties of connecting fitness trackers, another reason for leaving out these devices is the idea of “Neues Credo”. The users should consciously plan and reflect on their physical activity. The automation of these steps by using a fitness tracker does not follow the aftercare concept. Furthermore, a bias would arise in the intervention group between users with fitness trackers and those without.

The patients constantly evaluate their self-imposed targets at intervals similar to those in the paper-based exercise diary. Based on an algorithm in the app, the patients receive feedback from ReNaApp regarding their documented physical activities in comparison with the recommended time frame [40]. The time intervals for feedback are similar to those of the classic feedback letters. Consequently, ReNaApp takes over this task from the aftercare agents in the post-rehab period.

## Methods

### Eligibility criteria

All patients from our five partner clinics with a reliable diagnosis of breast cancer will be included in this study (C50.0–.9 und D05.0–.9). Moreover, the rehabilitants must be between 18 and 60 years old, own a smartphone and have adequate German language skills.

All rehabilitants who do not have a reliable diagnosis of breast cancer or who abandon rehab are excluded from the study. Other criteria for excluding participants are insufficient German language skills, missing declaration of consent and constraints in physical activity.

### Study design

The study is organized as a mixed-methods study [41] with different methodological components (sections A-D). The leader of the study is the Institute for Social Medicine and Epidemiology of the University of Lübeck. The recruitment takes place in five different rehabilitation clinics in Germany (AMEOS Reha Klinikum Ratzeburg, Klinik Graal-Müritz, Strandklinik Ostseebad Boltenhagen, Asklepios Klinik Am Kurpark Bad Schwartau, Sonneneck Nordsee-Fachklinik Wyk auf Föhr). The designated aftercare agents of each clinic are responsible for the screening of eligible patients within the first days of their rehabilitation. If applicable, the agents address the patients personally and give them the information sheets and the declaration of consent.

### A: quantitative measures

To examine the long-term effects of oncological rehabilitation with ReNaApp, the study is structured as a monocentric, quasi-randomized longitudinal study with three measurement points: at the beginning (t_0_) and end of rehabilitation (t_1_) and 12 months after rehabilitation (t_2_). All patients will complete a written questionnaire using standardized, validated instruments.

Following a sequential design, all rehabilitants who arrive in the rehab clinics belong to the control group (standard therapy and standard aftercare) until the desired number of cases is reached. After the recruitment of the control group, the rehab teams of all clinics are instructed in the handling of ReNaApp and the aftercare philosophy. The implementation of the aftercare intervention is accompanied by a fundamental change in the procedures and therapy regimens in clinics; therefore, recruitment of the control and intervention groups at the same time will not be possible. The later rehabilitants who fulfil the eligible criteria will be allocated to the intervention group. Based on preliminary interviews in rehab clinics, we assume that most of the rehabilitants own a smartphone and know how to use a mobile application.

#### Primary outcome

*Restrictions for social and work-related participation* will be captured with the IMET [42]. This validated questionnaire determines the impairment of persons with a chronic disease by the International Classification of Functioning, Disability and Health (ICF). The IMET imposes restrictions on participation in nine relevant dimensions in everyday life. The appraisal for each dimension is based on a scale from 0 to 10, and it is possible to analyse single items as well as the cumulative score. High scores implicate highly restricted participation. The IMET is a validated questionnaire for chronically ill patients [43, 44].

*Breast cancer-specific quality of life* will be measured with the EORTC-QLQ-BR23 [45]. The Quality of Life Questionnaire BR23 is an additional module from EORTC-QLQ-C30 that was developed for use in patients with breast cancer in different disease stages and with different types of therapy. The items include side effects from the systematic forms of therapy, such as arm symptoms, breast symptoms, body image, and sexual functions, and single items, such as sexuality, loss of hair, and future perspectives. All of these subscales can be ranked between 0 and 100 points. A higher score induces a better function and a higher quality of life. In the system subscales, a higher score signifies a higher level of symptoms and problems [45]. The QLQ-BR23 is a validated, reliable questionnaire used in numerous studies.

*Return to work* will be captured in a retrospective way with two single items. The rehabilitants are asked whether they returned to work after rehabilitation, and if so, how much time (in months) passed between rehab and return to work.

#### Secondary outcomes

After medical rehabilitation, a great number of patients return to work without major problems [46, 47]. To include the work-related effects from this study, further work-related outcomes will be considered.

*Work ability* will be measured with the Work Ability Score (WAS) of the Work Ability Index (WAI). This single item consists of work ability on a scale from 0 to 10, and a higher score indicates higher work ability. The WAS has been applied in numerous studies and has acceptable reliability and validity [48].

*Occupational stress* will be measured with the German version of SIBAR II and IV, and the overall burden and differentiated single burdens will be addressed [49].

The SPE scale in the German language considers the *subjective prognosis of gainful employment*. Three items estimate the occupational risk of the patients. Adding the single items to form a total score, the patients can be subdivided into two risk groups. This scale has been validated in a large sample and is also reliable. Thus, the items have a good ability to obtain the subjective prognosis of gainful employment [50, 51].

*Occupational changes* will be measured with single items, including job-preserving interventions and types of job loss [52].

Cancer-related *fatigue* will be measured by the EORTC-FA12, a questionnaire validated in a large international, multicentre study [53]. With the aid of twelve four-staged items (“not at all” to “very”), physical, emotional, and cognitive fatigue impairments are investigated. During a German study validating the questionnaire with breast cancer patients [54], a high consistency of the overall scale was observed; the subscale also shows good to high internal consistency.

*Depressive state* will be measured by the CES-D (Center for Epidemiological Studies Depression Scale, German version). The scale consists of 20 four-level scaled items regarding the presence and frequency of depressive symptoms. The evaluation is made according to a total score that can reach a maximum of 60 points. The higher the value is, the higher the expression of depressive symptoms. The CES-D is a reliable and valid questionnaire [55].

The *performance* in various areas of life will be assessed by three scales from 0 to 10 for the areas job, everyday life, and leisure. A high score implies high performance. The three single items are approved as a valid procedure for the determination of performance restrictions [52, 56].

The extent of *physical activity* will be assessed in connection with the questionnaire of the Federal Health Survey by Menski, 1998 [57]. In addition, the use of health promotion services will be measured by a questionnaire from the Quality Community for Medical Rehabilitation [52, 56].

The moderating variable is comorbidity [58], as well as risk factors and sociodemographic variables [59]. Table 1 shows the set of the core instruments to be used in the study.

**Table 1:** Core set of instruments used in the quantitative part of the study

Dimensions	Instruments	t_0_	t_1_	t_2_
Primary Outcome				
Restriction of Participation	IMET [42]	●		●
Disease-specific Quality of Life	EORTC-QLQ-C30 [60]	●	●	●
	EORTC-QLQ-BR23 [45]			
Return to Work	Single Items			●
Secondary Outcomes				
Ability to Work	Work Ability Score, WAS [61]	●		●
Occupational Stress	SIBAR, II and IV [49]	●		●
Subjective Prognosis of Employment	SPE-Scale [62]	●		●
Occupational Changes	Single Items from QGmR [52]			●
Fatigue	EORTC-FA 12 [54]	●	●	●
Depressive State	CES-D [55]	●	●	●
Performance in Various Areas of Life	QGmR [52]	●		●
Physical Activity	Federal Health Survey [57]	●		●
Use of Health Promotion Services	QGmR [52]	●	●	●
Moderating Variables				
Comorbidity	Single Items [58]	●		●
Living Habits	Single Items	●	●	●
Sociodemographic Data	Single Items [59]	●		●

t_0_ = baseline/right before rehabilitation; t_1_ = 3-week follow-up/right after rehabilitation; t_2_ = 12 months after t_1_

Outcomes that cannot change during rehabilitation will be measured in the beginning and regard only catamnesis. These include outcomes regarding everyday life and the domestic environment of the patients (social participation, efficiency and physical activity in daily life) or insignificantly changed outcomes, such as medically determined comorbidities.

### B: qualitative measures

#### Interviews and focus groups with patients, aftercare agents and physicians

Six rehabilitants will be interviewed by telephone at 3, 6 and 9 months post-rehab. From their personal point of view, they should assess the aftercare strategy, the use of ReNaApp, and the current status of the aftercare programme. At the time of catamnesis and written surveys, the interviewees will be asked about the utility of the app and their acceptance of and satisfaction with the use of ReNaApp. In addition, the patients will express their wishes and record them for further development or optimization of the mobile application and aftercare strategy. The interviewees will differ in age and education to assemble a heterogeneous group as much as possible [63].

At the end of recruitment, several focus groups will be carried out with the aftercare agents and involved physicians of the five clinics. These focus groups will discuss the feasibility and practicability in clinical practice, the optimization possibilities, and the level of patient support provided by the ReNaApp.

All interviews and focus groups follow a guideline. The guidelines and the results of the qualitative assessment will be presented and discussed within the project team and in the interdisciplinary expert forum “Arbeitsgruppe qualitativer Methoden” (AQUAM; Working group on qualitative methods). The interviews will take approximately 30 min, while the focus groups will be longer. With the approval of every participant, the interviews and focus groups will be recorded and transcribed word by word. The subsequent evaluation will be performed according to the qualitative content analysis.

### C: formative evaluation of the mobile application

Generating a high usability is the main focus during the development of ReNaApp. More than 10 patient representatives (breast cancer patients) will receive the developed prototypes of ReNaApp and execute exemplary tasks, i.e., documenting physical activity in the diary.

The interactions with the system (prototype) have to be evaluated by objective data (processing time and accuracy, human mistakes), subjective information about satisfaction and acceptance (e.g., QUIS [64], UTAUT [65]) and mental demand (NASA-TLX [66]). Furthermore, problems due to the interaction with the system need to be discussed. Positive evaluated aspects of the design will be integrated into the development of the overall system.

### D: health economic evaluation

The aim of the health economic evaluation of ReNaApp is to determine the cost-effectiveness of the motion-based aftercare programme for women with breast cancer using an app (intervention group: IG) against the cost-effectiveness of a standardized rehabilitation measure with standard aftercare (control group: CG). Quantitative methods will be used to identify which intervention is more beneficial from an economic perspective. In addition, qualitative methods will be used to gain insights about factors that may enable or hinder aftercare implementation in rehabilitation clinics.

The standardized implementation of health economic studies requires the consideration of various conceptual and methodological aspects.

#### Data basis

The basis of this analysis comprises data of the primary and secondary outcomes of the rehabilitation surveys at t0 and t2, supplemented by questions regarding service consumption and other benefit parameters. Standardized patient questionnaires will be used for assessment, as well as the costs of intervention implementation in the facilities by means of structured interviews with rehabilitation facilities’ staff.

#### Types of health economic analyses

Proof of the advantage of the new intervention (rehabilitation model “Neues Credo” plus rehabilitation aftercare app) over the standard intervention in use (standard rehabilitation plus standard aftercare) will be provided by cost-effectiveness analyses, in which the effect parameters will be included in natural units (e.g., return to work) and via standardized and weighted uniform utility values (e.g., quality of life) [67–69]. Pre-post changes will be compared for the IG and the CG. The differences are called incremental costs, i.e., incremental effects. The result is shown as the ratio of incremental costs and the respective incremental benefit parameter [67]. An unfavourable effectiveness ratio can be caused by both low clinical effectiveness and high costs. A clinically less effective measure can also have superior cost-effectiveness if it offers high savings potential compared to the alternative procedure.

In addition, so-called cost-utility analyses will be carried out based on quality adjusted life years (QALYs). Here, the intervention-induced change in health status will be evaluated with health-related quality of life (QoL) measures, e.g., QALYs. QALYs are interpreted as an index for lifetime gain and QoL - a QALY represents 1 year of life in optimal health [70]. The incremental costs per QALY allow comparison with other breast cancer treatment interventions (EORTC-QLQ-C30 and -BR23) and with other indications (SF12) [67, 71, 72].

#### Perspectives of the study

The selection of relevant cost components is derived from the study hypothesis and the respective study perspective. For all studies, it is recommended that the analysis be carried out from a societal perspective to present the relevant benefits and resource consumption at a macroeconomic level. Additionally, the results that are relevant for the cost bearers (here: pension insurance institutions) should be added to describe possible divergences between the societal and cost bearers’ perspectives. The perspective of the patients is taken to identify possible influencing factors regarding consumption behaviour. Depending on the perspective, different exclusions are made for the cost and benefit parameters [67].

#### Cost parameters of the study

In health economic evaluations, a distinction is made between direct and indirect costs. By definition, direct medical and non-medical costs include the consumption of resources by health care sectors and patients or their social environment that are directly attributable to the disease or impairment and valued in monetary units.

For the ReNaApp study, the expected cost savings for both study groups from reduced service use in the 12 months after rehabilitation compared to the 12 months before rehabilitation (= net costs) will be determined. The difference between CG net costs and net costs, including the additional costs from the use of the new aftercare app (IG), will yield incremental costs.

All patients will document volume data of the utilization of services in the questionnaires.

Due to the lack of market prices, the evaluation of this database will be mainly based on prices administered by the state or agreed upon by association [67–70, 73]. The recommendations for determining valuation rates in health economic evaluation [74] will be considered as much as possible and supplemented by currently published billing approaches, e.g., [75–77].

To fully determine the costs from a societal perspective, co-payments, deductibles or other forms of co-payment to be made by patients, as well as costs incurred outside the social insurance system, (e.g., in the form of patients’ own contributions for non-reimbursable health care services and support services provided by the social environment), will also be included.

Indirect costs reflect the loss of value added due to the illness, valued in monetary units. From a pension insurance perspective, expenditure on rehabilitation and aftercare represents investments in maintaining earning capacity. To this extent, benefits for temporary and permanent reduction in earning capacity should be highlighted here. Costs arising from periods of incapacity to work on the part of the health insurance funds (sick pay) and employers (continued wage payments) and income losses from reduced working hours without financial compensation by the social insurance funds, e.g., reduced earning capacity pensions for female patients, are further cost items to be included. From a societal perspective, lost social security contributions also need consideration. In addition to these work- and employment-related productivity losses, ReNaApp accounts for reductions in performance in private life (e.g., housework, leisure time, voluntary work) to complete the patient perspective.

Two valuation approaches - the human capital approach (HCA) and the friction cost approach (FCA) - are available for determining the costs associated with the performance and productivity reductions. Both reflect the societal perspective, but they account for productivity losses differently. The controversial discussion about the two methodological approaches has not been resolved, so the recommendation to apply both approaches and examine the different outcome effects in sensitivity analyses is followed [67, 68]. Both approaches tend to neglect non-work activities (household, child-rearing, voluntary work, leisure time, etc.). Since it is to be expected that a significant proportion of the ReNaApp target population is already of pensionable age, the limited recording of indirect costs for the periods up to retirement can constitute discrimination. In this respect, a sensitivity analysis is additionally carried out based on the average remaining life expectancy [78].

For the assessment of the loss of value added due to sickness-related absence from work, the average gross annual income from dependent employment for the total working population [74] is applied to obtain results independent of indications.

#### Investment costs

Regarding the inclusion of intervention costs in health economic evaluation, different positions are held in the literature (see [67]). For the ReNaApp study, therefore, intervention costs are not included in the cost-effectiveness analysis or in the cost-utility analysis. They are used exclusively to determine a possible break-even point for the minimum number of patients necessary to cover the costs of the developmental intervention.

### Data management

#### Patient documentation

Study participants will be uniquely organized and documented in all clinics through an automated documentation template. All aftercare agents will be briefed on the template before recruitment.

The included study participants will receive an identification number (ID) to pseudomize their personal data. The first number serves the classification of the intervention and control groups. The subsequent numbering corresponds to the number of addressed participants. Personal data will be only documented in an independent Excel file; by the ID, an appropriate linkage occurs. The data will not be sent to the ISE until the personal contents are deleted. This collection only serves the standardized documentation in the clinics. The data from the questionnaire will be collected and kept apart from the ISE.

### Data entry and control

In the context of the quantitative part of the study, (A) data from the written survey will be captured by the acquisition software TeleForm®. The answers from the questionnaires will be scanned by this software and finally transmitted directly into SPSS 22.0. The unrecorded answers will be completed by a student assistant under the guidance of a scientific employee.

### Data monitoring

This study does not contain drugs; ReNaApp investigates the health benefits of an aftercare programme fitted to the individual abilities of the patients. Therefore, no harmful or undesirable events are to be expected.

Our study is accompanied and advised by a data and safety monitoring committee. An interim analysis is not planned.

### Case number calculation/sample size

According to the time and primary outcomes, we expect that both groups (control and intervention groups) will similarly improve until the end of rehab. In the following period, the effects in the CG will approach the starting level before rehab, while the positive changes in the IG will stabilize and even increase slightly. We assume that the intervention group will return to work with higher likelihood and earlier than the CG.

Regarding the needed sample size, the primary outcome *restriction in participation* (IMET [42]) will be involved. For this purpose, we have results from our own studies about the influence of “Neues Credo”. To determine the effect of data on the Quality Community for Medical Rehabilitation (QGmR, indication oncology) [52], the controlled trial of “Neues Credo” [14] and the feasibility study of “Neues Credo” [15] were taken into account.

The QGmR data from oncological patients who followed a standard rehab and standard aftercare did not show an effect (ES = 0.05) on participation after a four-month follow-up. The IG participation in the controlled trial of “Neues Credo” showed an effect intensity of ES = 0.42 in a 12-month follow-up, and the control group showed an effect intensity of ES = 0.06. Psychosomatic rehabilitants reached an effect intensity of ES = 0.53 for participation after a four-month follow-up in the feasibility study of “Neues Credo”.

Calculating the sample size, we assume clinically relevant and positive effects (pre-catamnesis-ES = 0.40) in *social and work-related participation* (IMET [42]) in the IG after a 12-month follow-up. Compared to the intervention group, the control group does not show any effects to the point of catamnesis. The other two primary outcomes are expected to show comparable effect intensity [79–81]. To demonstrate differences between the IG and CG after a 12-month follow-up, we require a magnitude of ES = 0.40 by double-sided testing with α = 1.6% (Bonferroni correction for three primary outcomes) with a power of 80%. That is why the intervention and control groups need a net sample size of *n* = 235 for each. We expect a dropout rate of 30% from patients with oncological rehab (according to experiences in QGmR oncology). To analyse each group of n = 235 patients, we must initially recruit 336 participants for the study; i.e., every rehab clinic has to recruit 112 participants (56 participants in each group).

With propensity score matching (PSM), it is possible that not all participants will have a match despite the use of a caliper. The equalization of these matching failures justifies a 10% higher sample size; thus, 740 patients must be recruited (*n* = 370 for IG and CG). Taking into account that 30% of those asked will refuse to participate in the study, 528 patients of each group must be addressed to participate in the study. In summary, each clinic will address *n* = 176 patients, 88 patients in each group. Because the study does not examine differences between the clinics, the rehabilitants of each clinic will be considered as one group at the end of recruitment.

### Statistical methods

#### Quantitative analysis

The longitudinal data will be analysed by variance analysis with repeated measurements; the nominal and ordinal scaled parameters will be evaluated by analysis of contingency tables; the subgroup analysis will be carried out with qui-square tests and t-tests.

The primary outcome *return to work* will be retrospectively captured with a questionnaire after 12 months. The rehabilitants will provide information regarding whether they returned to work after rehab and how much time passed between rehab and the return to work. The time until the return to work is collected in models of survival analysis for discrete times. The entire analysis will be carried out with SPSS 22.0.

#### Qualitative analysis of the interviews and focus groups

The interviews and focus groups will be evaluated by qualitative content analysis [63, 82–84]. The program MAXQDA 12 accompanies the evaluation. The transcripts will be reviewed for relevant topics and interesting content to finally develop a category system. The main topics will be deduced from the research questions and the interview guideline; subcategories will be induced from materials, for example, developed by subsumption [82]. To test the category system first, an independent test coding will be performed by two scientists; afterwards, the categories and definitions will be modified. The whole coding process will be developed in the form of a consensual coding [84], i.e., the transcripts will be independently coded by two scientists. Last, the coding will be merged to develop a consensus.

## Discussion

According to the wishes of oncological patients, this intervention enables a supportive, home-based, and need-based aftercare programme.

The regular practices of physical activity have high curative potential for people with chronic and degenerative diseases [85]. Against the background of demographic change, the number of chronically ill people increases; thus, more support in carrying out physical activity is necessary. Furthermore, in the future, almost all rehabilitants will own a smartphone, making possible the widespread use of ReNaApp.

### Innovation factor

Currently, no aftercare programme for oncological patients exists. Despite the legal situation, oncological patients need to continue exercising after rehabilitation. However, the conditions at home make the realization of recommendations for exercise difficult despite the increased motivation though rehab.

Nevertheless, the aftercare concept “Neues Credo” is a valid strategy for chronically ill people to engage in more physical activity. Developing this strategy according to the growing importance of digitization, it is necessary to modify the concept from paper-based materials into a mobile application. Finally, digitalization offers new solutions for the healthcare sector.

Most of the patients who live in rural areas are disadvantaged regarding specific aftercare services. A mobile application offers the opportunity of an equal aftercare programme to all patients.

Last, this concept develops through a participative process. Focus groups comprising breast cancer patients and conversations with members of breast cancer self-aid groups supported a high interest and need for this application. In addition, the whole study processes will be accompanied by patient representatives to safeguard the user perspective and individual expectations and wishes. This strategy aims to exploit the potential of ReNaApp. The app can positively affect permanent integration in everyday life. Under this consideration, we expect the rehabilitants to indicate a high usability and very good acceptance regarding ReNaApp.

### Immediate expected results of the project

The use of ReNaApp for 12 months leads to clinically relevant and positive effects (pre-post-ES > 0.40) in different primary outcomes after rehab. In addition to a significant increase in social and work-related participation in the IG, breast cancer patients in the IG return faster and more often to work than patients following the standard aftercare programme. This is accompanied by lower indirect costs in the IG. Furthermore, the quality of life increases.

The extent of restriction in social and work-related participation and the quality of life of the control group are comparable to the conditions of the intervention group at the beginning of rehab. Additionally, the participants in the control group returned to work less often and later than participants in the intervention group.

Compared to the CG participants, IG participants using ReNaApp show significant positive effects in all secondary outcomes 1 year after rehab. This includes less physical, psychological and occupational stress as well as a better prognosis of employment and work ability.

Last, the intervention group shows differences compared to the control group regarding a significantly stronger extent of physical activity 1 year after rehab. Unlike the patients in the control group, the breast cancer patients in the intervention group more often engage in self-engaged fitness training or sports programmes though the ReNaApp; they practice significantly more endurance sports.

### Transferability of the project results to the everyday care situation

The aftercare concept “Neues Credo”, modified as a mobile application, aims to maintain the long-term effects of rehab of oncological rehabilitants. Focussing on high individual responsibility, the strategy also targets greater empowerment and self-control of the rehabilitants. During rehab, the participants are trained in these strategies. Afterwards, the rehabilitants transform into their own motivators and the experts of their own aftercare.

The advantage of the new strategy is the time and financial relief for aftercare agents gained because of an integrated self-control and motivational feedback tool. The only temporal expenditure for the clinics regards sending motivation letters to the patients and, if necessary, providing contact in case of questions.

The intervention ReNaApp will be implemented and evaluated in five oncological rehab clinics for breast cancer patients in Germany. In general, it is possible to transfer the intervention to other clinics. Undoubtedly, the developed mobile application could be adapted and expanded for other diseases. Thus, this app could support all people needing support in home exercise.

To secure the transfer to mobile technology, information and materials are compiled for cost units and rehab clinics.

### Limitations

First, it is possible that the disease worsens during and after rehabilitation. In this scenario, patients could contact their aftercare agents and review the personal aims and plans for physical activity and adapt them to the new situation.

In the technical part of the study, the risk of failure of the mobile application exists. In this case, we have the chance to consult our experts of IMIS and ITM to, e.g., provide new updates for the application.

Personal data will never be transferred from the application to the study leader or aftercare agents. ReNaApp keeps the information, statistics and documentation private.

To ensure equal treatment for the IG and CG, all characteristics of the participants (e.g., age, education) and the disease (e.g., disease severity, disease status, and comorbidity) will be documented and compared between the groups. If differences arise between the two groups, they will be comparably integrated in the analysis (e.g., as covariates in multivariate methods or by propensity score matching). Based on our present knowledge, we assume that structural changes in the context of this project are very unlikely.

## Data Availability

The datasets generated and/or analysed during the current study are not publicly available due to the data protection regulations and declarations of consent of the study participants but are available from the corresponding author on reasonable request. The declarations of consent do not include the release of the data for external analyses. A sample declaration of consent is available from the corresponding author on request. The study results are published and thus made available to all those interested in the study. It is not planned to make the individual participant data (IPD) available. The study protocol, the dataset at the participant level and the statistical code for generating the results will also not be made publicly available.

## Abbreviations

AQUAM: Arbeitsgruppe qualitativer Methoden is a German Working group on qualitative methods
CG: Control group
FCA: Friction cost approach
HCA: Human capital approach
ICF: International Classification of Functioning, Disability and Health
ID: Identification number
IG: Intervention group
IPD: Individual participant data
PSM: Propensity score matching
PWA: Progressive web application
QALYs: Quality adjusted life years
QoL: Quality of life
RCT: Randomized clinical trials
